# CSII treatment as an option in high-risk pregnant patients with type 1 diabetes

**DOI:** 10.1186/1758-5996-7-S1-A84

**Published:** 2015-11-11

**Authors:** Íkaro Soares Santos Breder, Arnaldo Moura Neto, Jéssica Castro Vasconcelos, Maria Caroline de Azevedo e Souza Netto, Denise Engelbrecht Zantut Wittmann, Walter José Minicucci

**Affiliations:** 1 Universidade Estadual ee Campinas, Campinas, Brazil

## Background

Studies highlight that continuous subcutaneous insulin infusion (CSII) during pregnancy leads to better glycemic control and lower glycated hemoglobin (HbA1c) without an increase in hypoglycemic episodes or diabetic ketoacidosis. Poor glycemic control can lead to birth defects, miscarriages, growth abnormalities and stillbirth. However, how to obtain better gestational outcomes is still debatable.

## Aims

Evaluate glycemic control in pregnant women with type 1 diabetes (T1D) in use of CSII, assessing the improvement of glycemic control and gestational outcomes.

## Materials and methods

This is a descriptive, single observer study carried out in a specialized endocrinology clinic. Eight pregnant patients with T1D in use of CSII and nine newborns were evaluated. Maternal variables analyzed were: age, diabetes duration, time of CSII Background during pregnancy, HBA1C before and during pregnancy; and newborn variables: intensive unit care (ICU) admission, neonatal hypoglycemia, prematurity, low birth weight and large for gestational age (LGA).

## Results

Mean age at pregnancy was 29.3 (±4.1) yrs. Mean diabetes duration before pregnancy was 11.9 (±5.6) yrs. Five of eight (62.5%) had an absolute contraindication to pregnancy. Three (37.5%) became pregnant already in use of CSII; one (12.5%) started CSII in the first trimester and four (50%) in the second trimester. All but one was subjected to cesarean delivery. None had diabetic ketoacidosis. There was a significant HbA1c decrease in the third trimester compared to pre-pregnancy values (6.8% vs 9.4%, p=0.028) and from the second to first trimester (7.1% vs 8.1%, p=0.043). There was one twin pregnancy. There were no stillbirths, miscarriages or congenital anomalies. Five of nine babies (55.6%) were premature, four (44.4%) were required ICU admission, five (55.6%) had neonatal hypoglycemia and three (33.3%) were LGA. Prematurity was more common in older mothers (p=0.029).

## Conclusion

As expected, CSII in pregnancy leads to a reduction in HBA1c without increasing the frequency of diabetic ketoacidosis, even in high-risk patients or those with previous poor compliance. The relatively good Results achieved in this group of patients with previous adverse conditions for pregnancy could related to the attainment of better glycemic control with CSII. Therefore, we believe that CSII treatment could be an option for patients that start pregnancy with a poor prognosis.

